# Impacto de la mutación T315I en el pronóstico de la leucemia mieloide crónica

**DOI:** 10.1515/almed-2024-0172

**Published:** 2024-11-27

**Authors:** Bushra Kaleem, Sadaf Shahab, Tahir Sultan Shamsi

**Affiliations:** Department of Clinical Research, National Institute of Blood Disease and Bone Marrow Transplantation, Karachi, Pakistan; Department of Clinical Haematology, National Institute of Blood Disease and Bone Marrow Transplantation, Karachi, Pakistan

**Keywords:** *BCR-ABL*, leucemia mieloide crónica (LMC), progresión de enfermedad, supervivencia general, resistencia, mutación T315I

## Abstract

**Objetivos:**

Las mutaciones dominio quinasa *BCR-ABL1* son una de las principales causas de resistencia a los inhibidores de la tirosina quinasa (ITK) en la leucemia mieloide crónica (LMC), siendo la mutación T315I la más resistente a tratamiento. El objetivo del presente estudio es determinar la frecuencia de T315I y su impacto en el pronóstico de la enfermedad, en términos de progresión de enfermedad y supervivencia.

**Métodos:**

Se clasificó como pacientes sin respuesta a tratamiento a aquellos pacientes con respuesta clasificada como “en advertencia” o que no mostraron respuesta completa a tratamiento con ITK, según los criterios de LeukemiaNet (ELN). La mutación T315I se detectó mediante el sistema de mutación refractario a la amplificación por PCR (ARMS-PCR), cuyo resultado fue posteriormente validado mediante secuenciación. Se realizó un seguimiento de 96 meses para observar el impacto de la mutación en el pronóstico de estos pacientes.

**Resultados:**

De los 102 pacientes que no respondieron a tratamiento, la mutación T315I fue detectada en el 21,6 %, con mayor preponderancia entre las mujeres. El 59 % de los pacientes portadores de la mutación presentaron un riesgo basal bajo por la escala Sokal. El 58,8 % de los portadores desarrollaron progresión de la enfermedad a la fase blástica. La supervivencia general (periodo de estudio: 96 meses) de los portadores de la T315I fue del 81,8 % de los pacientes portadores. Los pacientes que progresaron a la fase blástica presentaron mayor probabilidad de ser portadores de la mutación T315I.

**Conclusiones:**

Una respuesta subóptima o falta de respuesta a tratamiento con ITK indica el desarrollo de resistencia debido a la presencia de la mutación T315I o de otras mutaciones. La identificación temprana de esta u otras mutaciones ayudaría a reorientar el tratamiento del paciente.

## Introducción

La leucemia mieloide crónica (LMC) es un neoplasma mieloproliferativo causado por una translocación recíproca t(9;22) (q34.1;q11.2) que da lugar a un oncogén que codifica una oncoproteína de 210 kD (dominio quinasa) con actividad de proliferación celular aumentada [1]. El oncogén resultante, *BCR-ABL1*, es un factor diagnóstico distintivo en el 95 % de los pacientes con LMC.

El desarrollo de inhibidores de la tirosina quinasa (ITK) requirió un profundo conocimiento previo de la patofisiología de la proteína de fusión. Estos compuestos se diseñaron para inhibir específicamente esta proteína de fusión. El inhibidor de primera generación, Imatinib, junto con los ITK de segunda y tercera generación, modificaron la estrategia de tratamiento de la LMC. En el estudio internacional aleatorizado de interferón frente a STI571 (IRIS), Imatinib mostró efectos prometedores, con una supervivencia global (OS) del 83,3 % y una respuesta citogénica completa (CCyR) del 82,8 %. No obstante, alrededor del 20 % de los pacientes desarrolló resistencia a este tratamiento [2]. Estas mutaciones genéticas impidieron la unión de Imatinib, al interferir en el lugar de unión o alterar la configuración de BCR-ABL, dando lugar a una conformación con menor afinidad por el Imatinib [3]. De todas las mutaciones identificadas, T315I fue la más prevalente, ya que representó entre el 4 y el 19 % de la totalidad de las mutaciones que causan resistencia en los pacientes con LMC [4].

Con la introducción en 2006 del inhibidor ATP de BCR-ABL, Nilotinib, un ITK de segunda generación más competitivo, se lograron mejores resultados clínicos en casi el 50 % de los pacientes con resistencia primaria o secundaria al tratamiento con Imatinib [4, 5]. Así, se lograron respuestas citogenéticas y hematológicas notablemente mejores en todas las fases de la LMC [5]. Sin embargo, a pesar del desarrollo de las nuevas ITK, algunos pacientes continúan desarrollando resistencia a tratamiento, siendo la mutación T315I una de las principales causas, según los análisis mutacionales. Esta mutación genética resulta de la sustitución de la treonina por la isoleucina. En un estudio en 386 pacientes con LMC que progresó a la fase blástica, se observó que la mutación T315I es una de las mutaciones más prevalentes [6]. En otro estudio en 176 pacientes con LMC que recibieron tratamiento con ITK de primera o segunda generación que eran portadores de la mutación T315I, se observó que los pacientes con enfermedad en fase blástica fueron los que presentaron una menor tasa de supervivencia, con una media de solo cuatro meses, frente a los 22,4 meses de supervivencia global en los pacientes con LMC en fase crónica [7]. El presente estudio se realizó para evaluar el impacto de la presencia de la mutación T315I en pacientes con LMC en el pronóstico de la enfermedad, en términos de progresión de enfermedad y supervivencia.

## Materiales y métodos

Realizamos un estudio unicéntrico longitudinal en el Instituto Nacional de Enfermedades Hematológicas y Trasplante de Médula Ósea sobre el tratamiento de pacientes con LMC en tratamiento con ITK. El estudio fue aprobado por el Comité Ético local (NIBD/RD/159-41-2015). Se obtuvo el consentimiento verbal voluntario de los participantes en el estudio. Se identificó como pacientes sin respuesta a tratamiento a aquellos pacientes que desarrollaron resistencia primaria [esto es, que no mostraron respuesta hematológica completa (RHC) y/o respuesta citogenética significativa (MCyR) a los 3 y 6 meses respectivamente] o resistencia secundaria [progresión a enfermedad avanzada o pérdida de la respuesta lograda, paralelamente a un incremento de cinco a diez veces de las transcripciones *BCR-ABL*, determinada mediante reacción en cadena de la polimerasa en tiempo real estandarizada], siguiendo las recomendaciones de la ELN, transcurridos 12 meses desde el inicio del tratamiento [8]. Se reclutó de manera consecutiva a los pacientes sin respuesta, a cuyas muestras se les realizó un análisis mutacional para determinar la presencia de T315I mediante el Sistema de Mutación Refractario a la Amplificación por PCR (ARMS-PCR) empleando los cebadores y las condiciones de termociclado descritas en un estudio anterior [9]. La presencia de la mutación positiva se validó mediante le método de secuenciación de Sanger. Se realizó un seguimiento a los pacientes durante un amplio periodo de ocho años para determinar el impacto de la mutación en la progresión y el resultado clínico de la enfermedad, especialmente en relación a la progresión a la fase de aceleración o a crisis blástica y a la mortalidad, y evaluar la tasa de supervivencia global. Se excluyó a aquellos pacientes con otros neoplasmas mieloproliferativos u otras patologías hematológicas malignas, así como a los menores de 18 años.

### Análisis estadístico

Todos los análisis estadísticos se realizaron con SPSS Versión 26.0. Tras evaluar la normalidad en la distribución de los datos mediante la prueba U de Shapiro-Wilk, se calcularon la mediana y los rangos intercuartílicos en el caso de las variables cuantitativas, y las frecuencias y porcentajes en las variables cualitativas. La significación en las asociaciones se analizó mediante la prueba exacta de Fisher o de Chi cuadrado (según corresponda). Así mismo, se evaluó la probabilidad de presentar la mutación en relación a diferentes factores. Un valor de p<0,05 se consideró significativo. La supervivencia global se calculó mediante el análisis de supervivencia de Kaplan-Meier.

## Resultados

En la fase de reclutamiento, se identificó a 102 pacientes sin respuesta a tratamiento. De estos 102 pacientes, 22 (21,6 %) eran portadores de la mutación T315I (Tabla 1). De los 22 portadores de la mutación T315I, el 54,5 % (12/22) eran mujeres, mostrando así una mayor prevalencia de la mutación en pacientes de sexo femenino. La mediana de edad (RIC) de los portadores de la mutación fue de 37,5 años (31.5–46.5 años), una edad significativamente menor a la del grupo de los no portadores de la mutación, que fue de 45,2 años (39,5–60,3 años). En el análisis retrospectivo de los portadores de la mutación T315I, en el que se determinó el índice en la escala Sokal, observamos que la mayoría de los portadores, más concretamente el 59,1 %, habían sido clasificados como pacientes de bajo riesgo, seguidos de aquellos con riesgo intermedio (31,8 %; 7/22), y riesgo elevado (9,1 %; 2/22). Se observó una tendencia similar en el grupo de no portadores, siendo la mayor parte del grupo pacientes de bajo riesgo. El porcentaje de pacientes con resistencia primaria fue mayor en el grupo de portadores de la mutación (5/22; 22,7 %) que en el grupo de no portadores (11/80; 13,8 %).

**Tabla 1:** Asociación de diversos factores con la presencia de la mutación.

	Mutación		
Variables	Presente (n=22), n (%)	Ausente (n=80), n (%)	Valor p
**Género**			
Masculino (n=61)	10 (45,5)	51 (63,8)	0,145
Femenino (n=41)	12 (54,5)	29 (36,2)	
**Resistencia**			
Primaria (n=16)	5 (22,7)	11 (13,8)	0,328
Adquirida (n=86)	17 (77,3)	69 (86,2)	
**Índice de riesgo de sokal relativo**			
Bajo (n=65)	13 (59,1)	52 (65,0)	0,792
Intermedio (n=30)	7 (31,8)	23 (28,8)	
Alto (n=7)	2 (9,1)	5 (6,3)	
**Progresión de la enfermedad**			
Sin progresión (n=40)	5 (22,7)	35 (43,8)	0,051
Con progresión a FA^a^ (n=35)	7 (31,8)	28 (35,0)	
Con progresión a CB^b^ (n=27)	10 (45,5)	17 (21,2)	
**Estado de supervivencia**			
Vivo (n=89)	18 (81,8)	71 (88,8)	0,470
Fallecido (n=13)	4 (18,2)	9 (11,2)	

^a^FA, fase acelerada; ^b^CB, crisis blástica.

**Figura 1:**
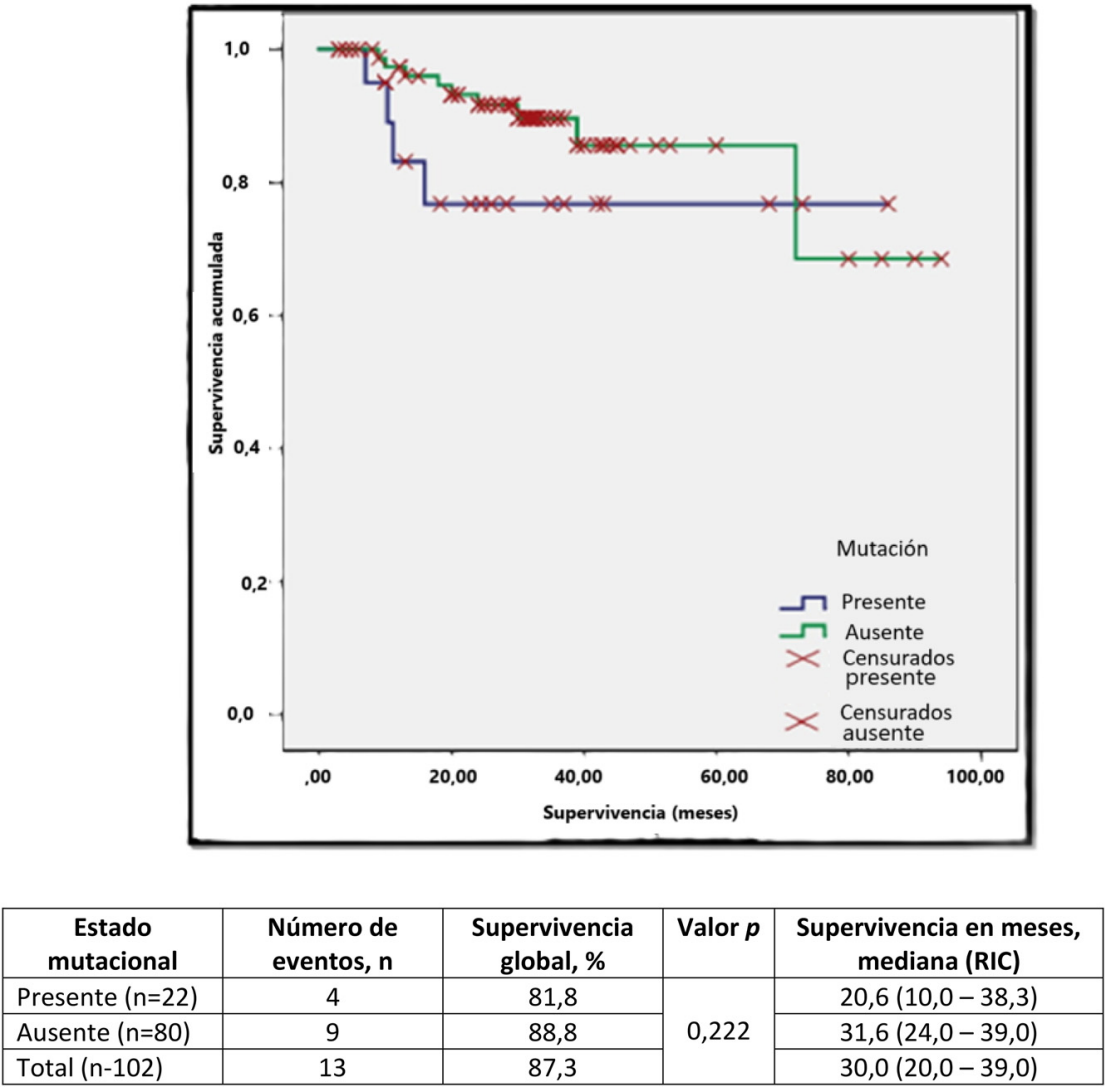
Análisis de supervivencia de Kaplan-Meier en función de la presencia de la mutación T315I.

Se observó progresión de la enfermedad, entendida como el avance a la fase acelerada o crisis blástica, en 17 (77,3 %) portadores de la mutación, de los cuales el 58,8 % (10/17) habían progresado a crisis blástica. Por otro lado, el 56,3 % (45/80) de los no portadores presentaron progresión de la enfermedad, de los cuales únicamente el 37,8 % (17/45) progresaron a la fase de crisis blástica. En la Tabla 1 se puede observar la asociación entre la presencia o ausencia de la mutación T315I y diversos factores. La supervivencia global observada en un periodo analítico de 96 meses, fue del 81,8 % (tiempo) en los portadores de la mutación T315I (Tabla 1 y Figura 1), frente a una supervivencia del 88,8 % en el grupo de no portadores de dicha mutación. En relación a la supervivencia (meses), la mediana de los portadores fue comparativamente inferior a la de los no portadores (20,6 meses frente a 31,6 meses). En la Tabla 2 se muestra la distribución de los pacientes en función del tratamiento recibido, la presencia o ausencia de mutaciones y si el paciente estaba vivo o no en el momento del análisis. Se observó que los portadores de la mutación recibieron Imatinib (n=5) o Nilotinib (n=17), sin que se realizara ningún cambio de tratamiento, mientras que los no portadores recibieron el tratamiento de primera línea (Imatinib=10 y Nilotinib=51) y un cambio posterior de tratamiento (cambio de Imatinib a Nilotinib=15 y de Nilotinib a Imatinib=4). Con respecto a la supervivencia de los portadores en función de la mutación y el ITK recibido, únicamente se observó mortalidad entre aquellos que recibieron Nilotinib (n=4/17), no habiendo fallecido ninguno de los que recibió Imatinib (n=0/5). Así mismo, sólo se observó mortalidad en el grupo de los no portadores que recibieron Imatinib (n=1/10), Nilotinib (n=6/51), y Imatinib con cambio a Nilotinib (n=2/15), no habiéndose producido mortalidad en el grupo con cambio de Imatinib a Nilotinib (n=0/4). De los 22 pacientes portadores de la mutación, solo cuatro recibieron un trasplante alogénico de células madre, de los cuales tres expiraron por causas relacionadas con el trasplante (trasplante frente a enfermedad del receptor-3), mientras que un paciente permanecía vivo y libre de enfermedad en el momento del análisis.

**Tabla 2:** Distribución de los pacientes en función del tratamiento recibido, el estado de supervivencia y el estado mutacional.

Mutación	Estado	TKI			
		Imatinib (n=5)	Nilotinib (n=17)	Imatinib a Nilotinib (n=0)	Nilotinib a Imatinib (n=0)
Presente (n=22)	Vivo (n=18)	5 (100,0)	13 (76,5)	**-**	**-**
	Fallecido (n=4)	0	4 (23,5)	**-**	**-**
		**Imatinib (n=10)**	**Nilotinib (n=51)**	I**matinib a Nilotinib (n=15)**	**Nilotinib a Imatinib (n=4)**
Ausente (n=80)	Vivo (n=71)	9 (90,0)	45 (88,2)	13 (86,7)	4 (100,0)
	Fallecido (n=9)	1 (10,0)	6 (11,8)	2 (13,3)	0

TKI, tyrosine kinase inhibitors.

En la Tabla 3 se muestra la probabilidad de ser portador de la mutación en función de diferentes variables. Como se puede observar, los pacientes en fase blástica tenían una probabilidad significativamente mayor de ser portadores de la mutación T315I (valor p = 0,02).

**Tabla 3:** *Odds ratio* de la presencia de la mutación T315I para distintos parámetros.

Variables	Razón de probabilidades* (odds ratio)*	Valor p	IC 95 %
**Género**			
Masculino	Ref.		
Femenino	2,110	0,125	0,812–5,484
**Resistencia**			
Primaria	Ref.		
Adquirida	1,845	0,310	0,565–6,02
**Índice relativo en la escala de sokal**			
Bajo	Ref.		
Intermedio	0,821	0,711	0,289–2,328
Alto	0,625	0,598	0,109–3,592
**Progresión de la enfermedad**			
Sin progresión	Ref.		
Con progresión a FA^a^	0,571	0,380	0,164–1,996
Con progresión a CB^b^	0,243	0,023^c^	0,072–0,823
**Estado de supervivencia**			
Vivo	Ref.		
Fallecido	0,570	0,392	0,158–2,065

^a^FA, fase acelerada; ^b^CB, crisis blástica; ^c^diferencia estadísticamente significativa.

## Discusión

El desarrollo de Imatinib supuso una revolución en el tratamiento de la LMC, convirtiendo una enfermedad maligna letal en una patología tratable. Posteriormente, se evidenció que para que el tratamiento fuese efectivo y duradero, era necesaria una exposición prolongada al mismo. Entre los tratamientos de segunda línea, se encuentra el tratamiento con Imatinib a dosis más elevadas, los tratamientos con ITK de segunda generación o el trasplante alogénico de células madre [10]. Algunos ensayos de fase III en pacientes con diagnóstico reciente de LMC han demostrado que los ITK de segunda generación, como el Dasatinib y el Nilotinib, también son superiores al Imatinib, mostrando una CCyR más rápida y sustancial y mejor respuesta molecular, además de mejores tasas de supervivencia global [11, 12].

Sin embargo, y a pesar de los importantes avances clínicos logrados, la resistencia a los ITK sigue siendo un problema en el tratamiento de la LMC. Entre las posibles causas de la resistencia primaria se encuentran un metabolismo y/o el transporte diferencial del fármaco. Por otro lado, algunas posibles causas de la resistencia adquirida podrían ser las mutaciones KD *BCR-ABL*, la amplificación del gen de fusión *BCR-ABL1*, la sobreexpresión de los genes transportadores del fármaco y la sobreexpresión de tirosinas quinasas como SRC [13, 14]. Estudios indios indican que entre el 45 y el 50 % de los pacientes con resistencia a Imatinib son portadores de mutaciones [15, 16]. En un amplio estudio con datos de casi un millar de pacientes de diferentes partes de la India, el 41 % presentaban mutaciones [17]. Según un estudio, el porcentaje de portadores de mutaciones también depende del estadio de la enfermedad de los pacientes incluidos en el análisis. Así, los pacientes con enfermedad avanzada podrían presentar una mayor frecuencia de dichas mutaciones [15].

La mutación T315I es una de las mutaciones de susceptibilidad tumoral más frecuentes. Su tratamiento es complejo, dado que tanto Imatinib como los ITK de segunda generación, Dasatinib y Nilotinib, resultan ineficaces [18]. En consonancia con el estudio realizado en India, el presente estudio reveló que la mutación T315I estaba presente en el 21,6 % de los pacientes que no respondieron a tratamiento [17]. Dicha proporción fue superior al 11,8 % obtenido en un estudio italiano [19]. Por otra parte, la frecuencia observada en el presente estudio fue inferior a la obtenida en el estudio de Khair y col, que observaron la presencia de la mutación T315I en el 43,4 % de los pacientes de su cohorte de estudio [20]. La predicción del pronóstico de los pacientes se realizó mediante el índice de Sokal en el momento del diagnóstico. El índice de Sokal obtenido en nuestro estudio indicaba que el 59,1 % de los portadores presentaban un bajo riesgo, seguido de un riesgo intermedio (31,8 %), y un riesgo elevado (9,1 %) (Tabla 1). Tal como también observaron Hasford y col [21], la frecuencia de la mutación en nuestro estudio fue inversamente proporcional al índice de riesgo [21].

Los clones con mutaciones en su dominio quinasa podrían presentar características biológicas distintivas que podrían aumentar su susceptibilidad a la leucemia, frente a los clones sin mutaciones [22]. Además, diversos estudios han demostrado que los portadores de la mutación T315I normalmente presentan peores resultados clínicos que aquellos con otras mutaciones o que desarrollan resistencia a Imatinib por otras causas [23]. Así, un estudio realizado en China reveló que el 68,4 % (13/19) de los pacientes con LMC portadores de la mutación T315I no respondieron a la escalada de dosis de Imatinib y progresaron a una fase avanzada [24]. Este hallazgo concuerda con los resultados de nuestro estudio, ya que el 77,3 % (17/22) de los portadores experimentó progresión de la enfermedad (Tabla 1), el 58 % de los cuales progresó a la fase blástica. Así mismo, observamos que los pacientes con enfermedad en fase blástica tenían 0,24 veces más probabilidades de desarrollar la mutación (valor p: 0,023), frente a aquellos con enfermedad crónica o en fase acelerada (Tabla 2). Otro estudio llevado a cabo en Egipto mostró que la frecuencia de las mutaciones fue superior en las fases avanzadas, esto es, en las fases acelerada y blástica (10/11, 91 %), frente a la fase crónica (6/17, 35 %) lo que indica una relación causal entre la progresión de la enfermedad y el desarrollo de una mutación [25].

La supervivencia global (en un periodo de estudio de 96 meses) en el presente estudio en pacientes portadores de la mutación T315I fue del 81,8 % frente al 88,8 % en lo no portadores (Tabla 1). No obstante, no se observaron diferencias estadísticamente significativas en relación a la supervivencia y la presencia de la mutación. La supervivencia global documentada por Jabbour y col fue del 59,3 % en los portadores de la mutación T315I, frente al 61,6 % en los no portadores [23].

El porcentaje de portadores parece variar en función de la metodología empleada, esto es, la PCR alelo específica (sensibilidad: 0.01 %), la cromatografía líquida desnaturalizante de alto rendimiento (sensibilidad: 0,1–10 %) o la secuenciación (sensibilidad: 10–20 %) [9]. Por tanto, en comparación con otras técnicas, se podría considerar que la ASO-PCR es un método rápido y económico para la detección de la mutación. No obstante, dado que en todo método de detección la secuenciación es considerada una prueba confirmatoria, se debería recomendar realizar la secuenciación toda vez que la mutación haya sido inicialmente detectada mediante ASO-PCR. Esta es la línea de acción que empleamos en nuestro estudio, debiendo ser recomendada especialmente ante la presencia de la mutación T315I, que puede incrementar el potencial leucemogénico de *BCR-ABL*, siendo además resistente a todos los ITK de primera, segunda y tercera generación, excepto el Ponatinib, que no está disponible en muchos países.

Dasatinib y Nilotinib, dos ITK de segunda línea efectivos contra múltiples mutaciones importantes contra las que Imatinib resulta ineficaz, no afectaron a la mutación T315I en BCR-ABL. El ensayo de fase II Ponatinib Ph+ALL and CML (PACE) reveló que Ponatinib es eficaz, ya que se obtuvo una respuesta citogenética importante (MCyR) en el 56 % de los pacientes con LMC en fase crónica, y una respuesta citogenética completa (CCyR) en el 46 % de los pacientes con resistencia a Dasatinib o Nilotinib que eran portadores de la mutación T315I [26]. Sin embargo, dicha medicación no está disponible en Paquistán.

Una de las limitaciones del presente estudio fue la imposibilidad de monitorizar la respuesta citogenética y molecular de nuestros pacientes en puntos temporales predeterminados. Dado que Ponatinib aún no está disponible en Paquistán, a los pacientes portadores de la mutación T315I se les ofrece terapia citorreductiva o un trasplante de médula ósea, que es un procedimiento económico.

## Conclusiones

Siguiendo las recomendaciones de la ELN, el análisis mutacional resulta esencial cuando el paciente desarrolla resistencia primaria o secundaria. Antes de cambiar a un tratamiento con ITK de segunda línea, se debe comprobar el estado mutacional del paciente. La presencia de una mutación ayuda a diseñar el siguiente tratamiento. La monitorización periódica resulta crucial a la hora de superar la resistencia y optimizar la respuesta. Dado que la tercera línea de tratamiento, Ponatinib, aún no está disponible en Paquistán, a los pacientes portadores de la mutación T315I se les ofrece terapia citorreductiva o un trasplante de médula ósea. Así mismo, es necesario implementar adecuadamente el análisis mutacional antes de cambiar a otro ITK, con el fin de evitar que la exposición a los fármacos lleve a clasificar a los pacientes “en advertencia” o sin respuesta a tatamiento.
